# Postpartum Deterioration of Hemodynamics in a Case of Uncorrected Ebstein's Anomaly

**DOI:** 10.1155/2012/841571

**Published:** 2012-06-10

**Authors:** Masato Kimura, Shuhei Kakizaki, Seiichi Tazawa, Osamu Adachi, Yoshikatsu Saiki, Shigeo Kure

**Affiliations:** ^1^Department of Pediatrics, Tohoku University Graduate School of Medicine, Miyagi, Sendai 980-8574, Japan; ^2^Department of Pediatric Cardiology, Nagano Children's Hospital, Toyoshina, Nagano 399-8288, Japan; ^3^Division of Cardiovascular Surgery, Tohoku University Graduate School of Medicine, Miyagi, Sendai 980-8574, Japan

## Abstract

Ebstein's anomaly is a rare congenital cardiac malformation that is characterized by abnormalities of the tricuspid valve and right ventricle. Pregnancy is usually well tolerated unless cyanosis or arrhythmia develops. We report a case with Ebstein's anomaly, whose condition was asymptomatic before pregnancy but remarkably deteriorated down during the postpartum period, even though a successful pregnancy and cardiac surgery were achieved. Women with Ebstein's anomaly should be carefully assessed and may need to be advised to have corrective surgery prior to pregnancy even if they were asymptomatic.

## 1. Introduction

Ebstein's anomaly is a rare congenital cardiac anomaly [1]. The malformation consists of apical displacement of the tricuspid valve (plastering of the leaflets to the endocardium) with dilatation of the atrialized portion of the right ventricle. The common cardiac anomalies associated with the condition are atrial septal defect and accessory pathway (e.g., Wolff-Parkinson-White syndrome). The clinical spectrum is varied; patients may be asymptomatic throughout their lives or may need to undergo surgery in the neonatal period. In Ebstein's anomaly, pregnancy is usually well tolerated unless cyanosis or arrhythmia develops, yet there is an increased risk of prematurity and low birth weight [2]. We report here a patient with Ebstein's anomaly, whose condition was asymptomatic before pregnancy but remarkably deteriorated down to the level of NYHA functional class IV during the postpartum period, even though a successful pregnancy and cardiac surgery were achieved. It should be recognized that the postpartum period is also a risk stage for women with Ebstein's anomaly.

## 2. Case Report

**Figure 1:**
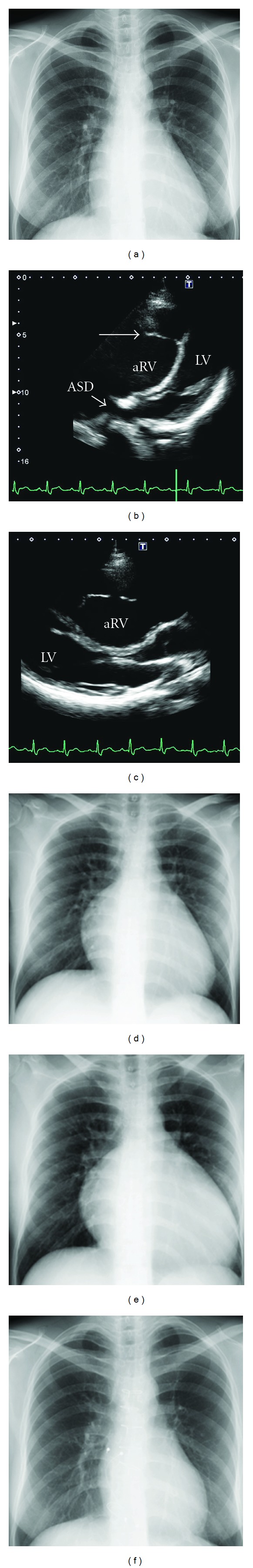
Chest X-rays (a, d–f) and echocardiographic images (b, c). Chest X-rays taken at gestational age (GA) 13 weeks (a), GA 33 weeks (d), 2 months postpartum (e), and three months after surgery (f). Cardiothoracic ratios were 54%, 63%, 70%, and 48%, respectively. Echocardiography performed at GA 13 weeks demonstrated apical displacement of the tricuspid valve (arrow) and an atrial septal defect (arrowhead) (b). Displacement of the interventricular septum by the atrialized portion of the right ventricle was observed both in systole (b) and diastole (c). aRV: atrialized right ventricle.

A 28-year-old primigravida was referred to our hospital at 13 weeks of pregnancy. She had been diagnosed with Ebstein's anomaly and an associated atrial septal defect at 3 years of age. She had been asymptomatic without medication. At age 25, she underwent cardiac catheterization for hemodynamic assessment anticipating a subsequent pregnancy. It revealed that her left arterial oxygen saturation (SaO_2_) was 93%, with good left ventricular function (Table 1). Surgical intervention was recommended due to severe tricuspid regurgitation and a remarkably enlarged, atrialized right ventricle. She declined to undergo surgery and became pregnant at the age of 28. At the first medical examination conducted at our hospital, she had no respiratory failure and had good percutaneous oxygen saturation (SpO_2_) of 93%. A chest X-ray confirmed cardiomegaly with a cardiothoracic ratio (CTR) of 54% (Figure 1(a)), and echocardiography revealed a dilated right atrium, a septal leaflet displaced by 50 mm from the tricuspid annulus, moderate tricuspid regurgitation (Carpentier's type C), and an ostium secundum atrial septal defect (Figure 1(b)). Left ventricular systolic function was preserved, though the interventricular septum was displaced by the enlarged, atrialized right ventricle (Figure 1(c)). At 22 weeks' gestation, her SpO_2_ decreased to 89%, and oxygen was administered by nasal cannula for cyanosis along with anticoagulation therapy (heparin) to prevent occult embolism. She was admitted at 27 weeks' gestation with dyspnea on exertion. Echocardiography revealed worsening tricuspid regurgitation and right side volume overload, but left ventricular systolic function seemed to be maintained. Although fetal growth was appropriate, elective cesarean section was performed at 33 weeks' gestation due to her respiratory failure and progressive desaturation with worsening cardiomegaly (Figure 1(d)). Her baby, weighing 1943 g (appropriate for date), was delivered with respiratory distress syndrome and required surfactant treatment. Pathological examination of the placenta was appropriate for gestational age. Two months after delivery, the mother's cardiac condition worsened to the level of NYHA class IV. A chest radiograph confirmed progressive cardiomegaly with a CTR of 70% and decreased pulmonary blood flow (Figure 1(e)). Urgent cardiac catheterization revealed SaO_2_ was 77% even with O_2_ administration, and a right-to-left shunt of 39%. Left ventricular function was severely impaired (Table 1). She underwent surgical repair with tricuspid valve replacement using a bioprosthetic valve (Epic Stented Tissue Valve, St. Jude Medical Inc, St Paul, MN) and ASD closure. A pacemaker was implanted (Reply DR, Sorin, Saluggia, Italy) due to complete AV block. During the postoperative period, diuretics (furosemide and aldactone), an angiotensin converting enzyme inhibitor (imidapril) and a beta-blocker (bisoprolol) were initiated. She was discharged in good hemodynamic condition with an ejection fraction of 57%, and she has remained well with an improved of CTR of 48% (Figure 1(f)).

## 3. Discussion

Presence of Ebstein's anomaly in a pregnant woman is not an indication for termination of pregnancy or a sufficient finding to advise against becoming pregnant [2]. In our patient, however, her heart condition deteriorated from NYHA class I before pregnancy to NYHA class IV in the postpartum period. It was difficult prior to pregnancy to predict such a striking hemodynamic change, and her hemodynamic condition deteriorated dramatically after delivery (Table 1). In general, plasma volume increases by as much as 50% in the third trimester [3], and we believe this is what induced dilation of the right atrium and atrialized right ventricle, resulting in an increased right-to-left shunt volume. The dilated right ventricle aggravated tricuspid regurgitation, which led to reduced pulmonary blood flow in the presence of a right-to-left shunt. Furthermore, left ventricular (LV) function was severely impaired in the postpartum period (Table 1). In a subgroup of patients with Ebstein's anomaly, LV dysfunction is innate [4]; however, this was not the case in our patient. On echocardiography, the ratio of transmitral Doppler early filling velocity to tissue Doppler early diastolic mitral annular velocity (E/e′) worsened from 4.8 at 20 weeks' pregnancy to 8.8 at two months after delivery. We suspect that the dilated, atrialized right ventricle interfered with LV diastolic function and that the superimposed severe cyanosis impaired both systolic and diastolic LV function. Moreover, two hemodynamic changes occur after normal delivery: the so-called autotransfusion of blood from the contracting uterus, which increases preload, and an increase in total vascular resistance, which increases afterload. Furthermore, as a physiological adaptation, augmented myocardial contractility declines after delivery [3]. These factors might have exacerbated heart failure in our case.

This case report suggests that women with Ebstein's anomaly should be carefully assessed and may need to be advised to have corrective surgery prior to pregnancy. Since significant hemodynamic changes occur after delivery, a patient with an uncorrected Ebstein's anomaly may be particularly susceptible to heart failure in the postpartum period.

## Figures and Tables

**Table 1:** Hemodynamic data on cardiac catheterization.

	aAo SpO_2_	Qp/Qs	L-R shunt	R-L shunt	mPAp	RA	LV/edp	LVEF	TR
	(%)		(%)	(%)	(mmHg)	(mmHg)	(mmHg)	(%)	
Antepartum (25 years old)	92.7	1.6	47	15	13	8	110/7	60.0	severe
Postpartum	76.8	1.2	49	39	21	17	105/18	12.6	severe
(29 years old)	(O_2_ 2L)								

aAo: ascending aorta, SpO_2_: percutaneous oxygen saturation; Qp/Qs: pulmonary/systemic blood flow ratio; mPAp: mean pulmonary artery pressure; RA: right atrium, edp: end-diastolic pressure; LV EF: left ventricular ejection fraction; TR: tricuspid regurgitation.

## References

[1] Freedom RM, Yoo S-J, Freedom RM, Yoo -J S, Mikailian H (2004). Ebstein’s malformation of the tricuspid value. *The Natural and Modified History of Congenital Heart Disease*.

[2] Donnelly JE, Brown JM, Radford DJ (1991). Pregnancy outcome and Ebstein’s anomaly. *British Heart Journal*.

[3] Silversides CK, Colman JM, Oakley C, Warens CA (2007). Physiological changes in pregnancy. *Heart Disease in Pregnancy*.

[4] Attenhofer Jost CH, Connolly HM, O’Leary PW, Warnes CA, Tajik AJ, Seward JB (2005). Left heart lesions in patients with ebstein anomaly. *Mayo Clinic Proceedings*.

